# Continuous immunotherapy beyond progression in clinical practice for small cell lung cancer

**DOI:** 10.1111/1759-7714.15308

**Published:** 2024-04-16

**Authors:** Ken Yamamoto, Taira Ninomaru, Hideaki Okada, Katsuya Hirano, Temiko Shimada, Akito Hata

**Affiliations:** ^1^ Division of Thoracic Oncology Kobe Minimally Invasive Cancer Center Kobe Japan

**Keywords:** beyond progression, immunotherapy, small cell lung cancer

## Abstract

In non‐small‐cell lung cancer, continuous immune‐checkpoint inhibitors (ICIs) beyond progression are often used in clinical practice. On the other hand, there is almost no data on whether the concept of continuous ICIs beyond progression can be adopted in small‐cell lung cancer (SCLC). We describe the effectiveness of continuous ICIs beyond progression in SCLC. Medical courses of SCLC patients treated with chemo‐immunotherapy were retrospectively reviewed at our hospital. The study included 36 patients with a median age of 73 years (range 46–83 years) who introduced chemo‐immunotherapy between September 2019 and December 2022. Atezolizumab and durvalumab in combination with platinum plus etoposide were administered in 24 and 12 patients, respectively. The overall response rate was 67% and the disease control rate was 86%. The median progression‐free survival and time to treatment failure (TTF) were 5.1 and 10.3 months, respectively. The median cycle of ICIs was 5 (range 1–42). The median overall survival was 13.6 months. ICIs were administered beyond progression in 14 (39%) patients: five were treated again with chemo‐immunotherapy and local ablative radiotherapy, four with local ablative radiotherapy and continuous ICIs, three with chemo‐immunotherapy, and two with continuous ICIs alone. TTF exceeded 12 months in 12 (86%) of the 14 cases, six of which were still on ICIs. Adverse events ≥grade 3 were observed in 21 (58%) patients. A notable TTF suggested a benefit of continuous ICIs beyond progression. The concept could be suitably adopted and provide a favorable prognosis in selected cases of SCLC that were previously regarded as an aggressive malignancy.

## Graphical abstract

The effectiveness of continuous immune‐checkpoint inhibitors (ICIs) beyond progression was suggested in clinical practice for small‐cell lung cancer. Continuous ICIs beyond progression were administered to the following three relapse types: type 1, sensitive relapse as systemic rapid progression; type 2, isolated relapse; and type 3, sensitive relapse as systemic indolent progression. Type 1 treatment was being re‐treated with chemo‐immunotherapy; type 2, local ablative radiotherapy and continuous ICIs; and type 3, continuous ICIs alone.

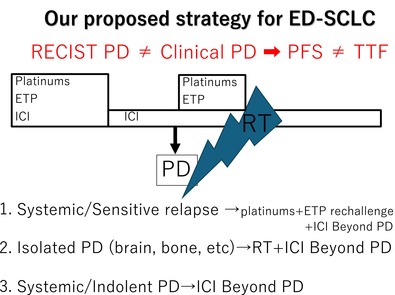

## INTRODUCTION

Lung cancer is the most common cause of cancer death in the world,^1^ and is classified into non‐small‐cell lung cancer (NSCLC) and small‐cell lung cancer (SCLC). Two‐thirds of SCLC patients already have extensive disease (ED) at diagnosis, and the prognosis for ED‐SCLC is generally poor.^2^

Recent advances in immunotherapy using immune checkpoint inhibitors (ICIs) has dramatically improved the prognoses for many types of cancers. The ICIs atezolizumab and durvalumab (antiprogrammed death‐ligand 1 antibodies) are now clinically available for ED‐SCLC, based on results of the IMpower133 and CASPIAN trials, which proved survival benefit in combination with chemotherapy.^3^, ^4^ However, long‐lasting survival appeared to be achieved in only 10%–15% of patients according to the survival curves of these trials. To improve the prognosis and maximize the effectiveness of ICIs, more pragmatic therapeutic strategy is required for ED‐SCLC.

In clinical practice for NSCLC, continuous ICIs after favorable response and tyrosine kinase inhibitors (TKIs) for cases with driver gene alterations are widely used beyond progression, and their clinical benefits have been reported.^5^, ^6^ However, to the best of our knowledge, there is almost no data on whether continuous ICIs beyond progression can be adopted in SCLC, therefore the aim of our study was to confirm that ICIs beyond progression improve the prognosis of SCLC.

## METHODS

This study was carried out at Kobe Minimally Invasive Cancer Center. We retrospectively reviewed electronic medical records to investigate the medical courses of SCLC patients treated with chemo‐immunotherapy. The study was conducted in accordance with the Declaration of Helsinki with the approval of the institutional review board. Treatment responses were assessed using Response Evaluation Criteria in Solid Tumors (RECIST), version 1.1. Progression‐free survival (PFS) was calculated from the beginning of chemo‐immunotherapy to the date of disease progression or death. Time to treatment failure (TTF) was calculated from the beginning of chemo‐immunotherapy to cessation of ICIs or death, regardless of disease progression. Overall survival (OS) was calculated from the beginning of chemo‐immunotherapy to death. PFS, TTF, and OS data are presented as Kaplan–Meier curves. Adverse events (AEs) were assessed with the National Cancer Institute Common Toxicity Criteria for Adverse Events (version 5.0). All statistical analyses were performed using JMP version 12.0 (SAS Institute Inc.).

## RESULTS

### Patient clinical characteristics

We evaluated 36 SCLC patients who had received chemo‐immunotherapy at our institute from September 2019 to March 2023. Patient characteristics are shown in Table 1. Their median age was 73 (range 46–83) years. Twenty‐two patients were male, and all patients had a smoking history. Twenty‐nine patients had an Eastern Cooperative Oncology Group performance status (ECOG PS) of 0–1. Thirteen patients had brain metastasis, eight had liver metastases, and eight had bone metastases. Twenty‐four patients received atezolizumab with carboplatin plus etoposide, and 12 received durvalumab with platinums plus etoposide. Seventeen patients were receiving first‐line treatment, 17 were being treated for recurrence after chemoradiotherapy, and two were receiving second‐line or later treatment.

**TABLE 1:** Patient characteristics.

Characteristic		Number (%)
Age (years)	Median (range)	73 (46–83)
Gender	Male	22 (61)
	Female	14 (39)
Smoking status	Ever	36 (100)
	Never	0 (0)
ECOG PS	0–1/ 2/ 3	29 (81)/6 (16)/1 (3)
Brain metastases	Yes	13 (36)
	No	23 (64)
Liver metastases	Yes	8 (22)
	No	28 (78)
Bone metastases	Yes	8 (22)
	No	28 (78)
Immunotherapy	Atezolizumab	24 (67)
	Durvalumab	12 (33)
Therapeutic lines	First‐line for ED	17 (47)
	Post‐CRT recurrence	17 (47)
	Second‐line or later	2 (6)

Abbreviations: CRT, chemoradiotherapy; ECOG, Eastern Cooperative Oncology Group; ED, extensive disease; PS, performance status.

### Efficacy

**FIGURE 1:**
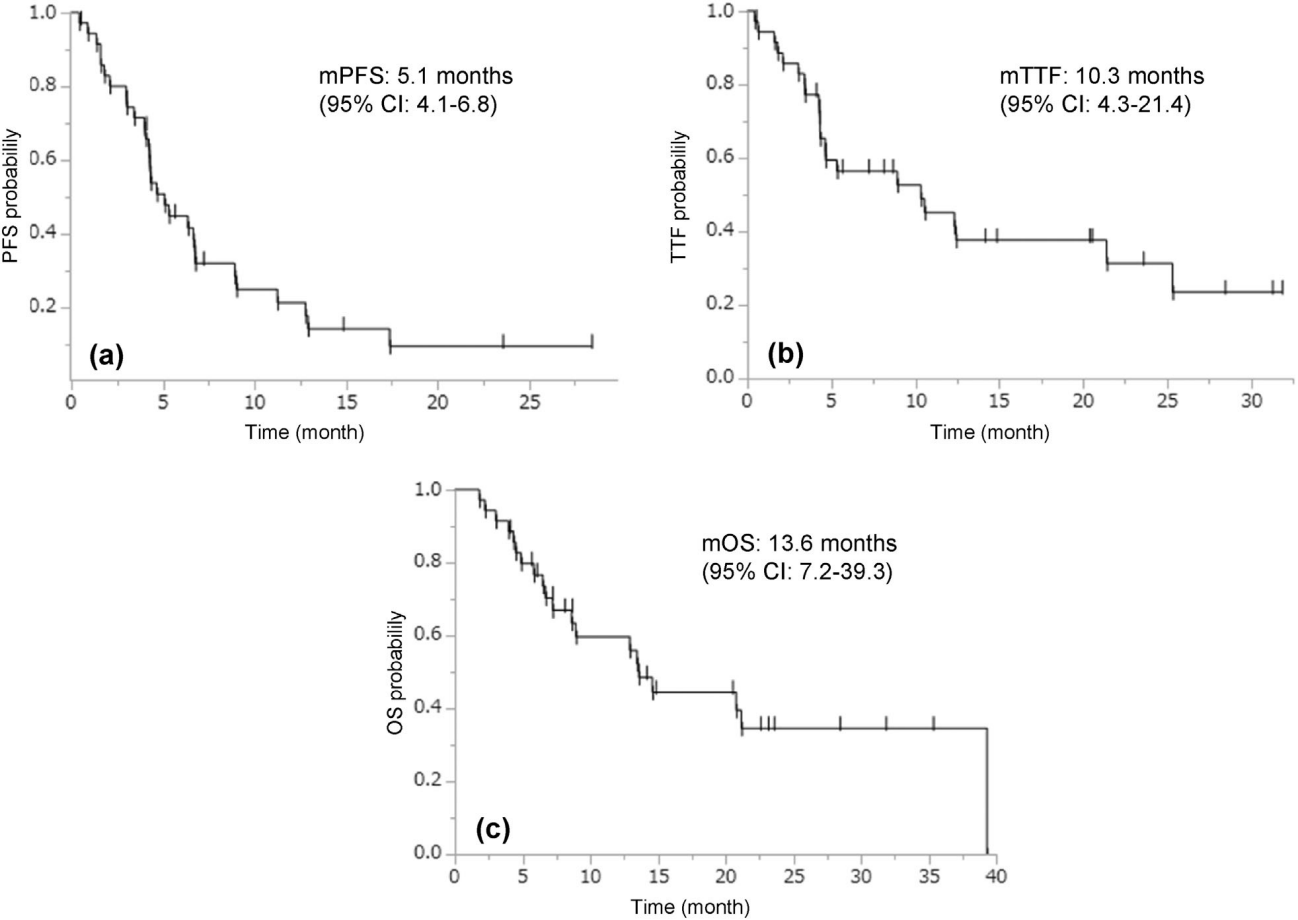
Kaplan–Meier curves of PFS (a), TTF (b), and OS (c). CI, confidence interval; mOS, median overall survival; mPFS, median progression‐free survival; mTTF, median time to treatment failure.

We observed one (3%) complete response, 23 (64%) partial responses, seven (19%) instances of stable disease, and five (14%) instances of progressive disease, resulting in an overall response rate (ORR) of 67% (95% confidence interval [CI] 49–81%) and a disease control rate of 86% (95% CI 70–95%). Twenty‐five (69%) patients completed chemo‐immunotherapy and moved to maintenance ICIs. The median cycles of ICIs was 5 (range 1–42). The median PFS and TTF were 5.1 (95% CI 4.1–6.8) months (Figure 1a) and 10.3 (95% CI 4.3–21.4) months (Figure 1b), respectively. The median OS was 13.6 (95% CI 7.2–39.3) months (Figure 1c).

**FIGURE 2:**
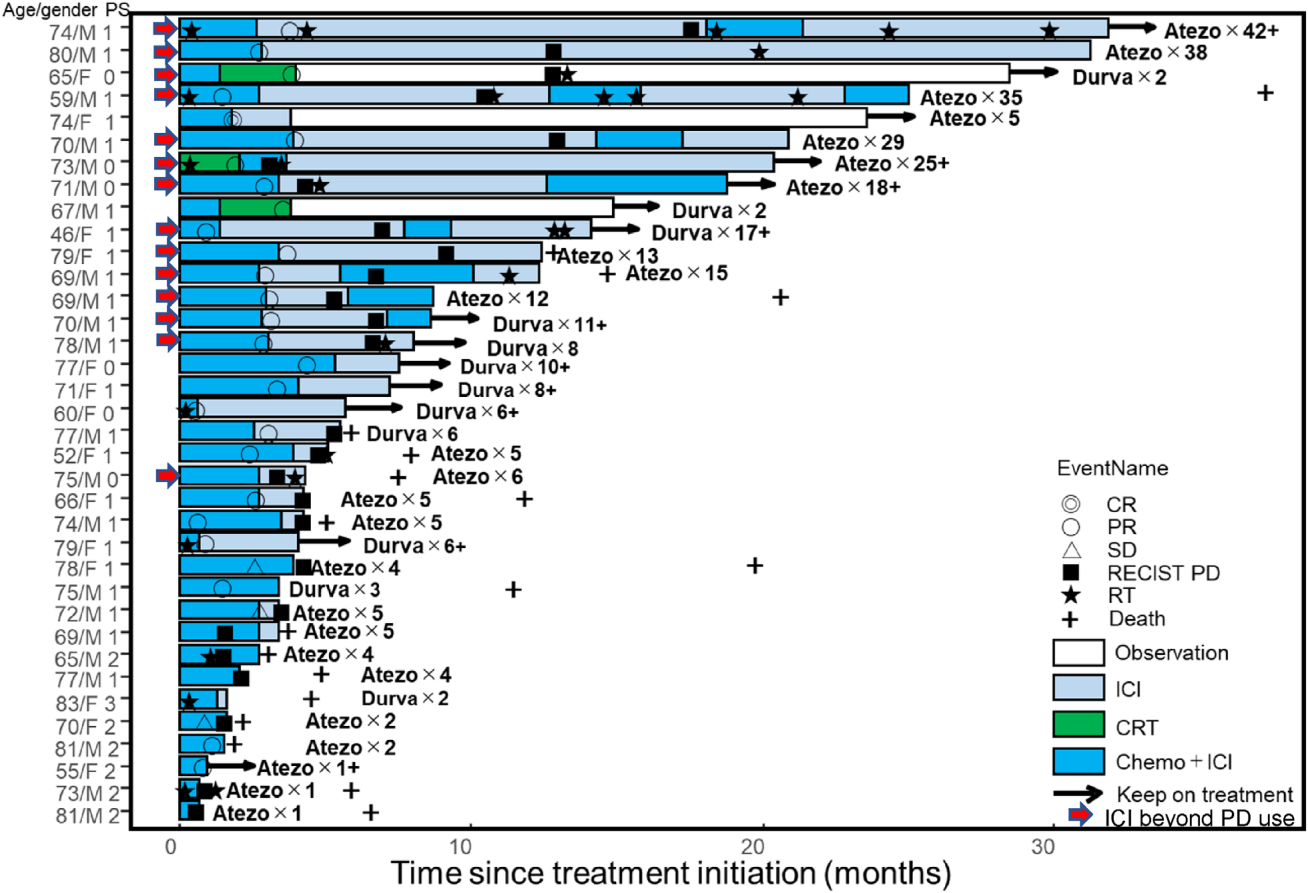
Swimmer plot shows the duration of TTFs in each case. Atezo, atezolizumab; CR, complete response; Durva, durvalumab; F, female; ICI, immuno‐checkpoint inhibitor; M, male; PD, progressive disease; PR, partial response; PS, performance status; RT, radiation therapy; SD, stable disease; TTF, time to treatment failure.

ICIs were administered beyond progression in 14 (39%) patients, of which five were treated again with chemo‐immunotherapy and local ablative radiotherapy, four with local ablative radiotherapy and continuous ICIs, three with chemo‐immunotherapy, and two with continuous ICIs alone. The durations of TTFs in each case are shown in a swimmer plot (Figure 2). TTF exceeded 12 months in 12 (86%) of the 14 cases, six of which were still on ICIs. Median PFS, TTF, OS, and the cycle of ICIs in the 14 patients receiving ICIs beyond progression were 6.7 (95% CI 3.0–11.2) months, 21.4 (95% CI 10.6–not reached) months, 39.3 (95% CI 12.9–39.3) months, and 16 (range 5–42), respectively.

### Safety

Grade 3 or higher AEs were seen in 21 (58%) patients: 19 (52%) neutropenia, four (11%) thrombocytopenia, two (6%) anemia, one (3%) febrile neutropenia, and one (3%) intestinal perforation. Grade 3 or 4 immune‐related AEs were observed in three (8%) cases: one (3%) hepatotoxicity, one (3%) diarrhea, and one (3%) arthritis. Treatment‐related death was exhibited in one (3%) case (grade 5 pneumonia) during induction chemo‐immunotherapy. There was no additionally unsafe signal during ICIs beyond progression.

## DISCUSSION

The ORR, PFS, and OS results of our study were comparable to previous clinical trials, despite including some elderly/poor PS patients.^3^, ^4^ What was intriguing in our study was the prolonged TTF (median 10.3 months) compared to PFS (median 5.1 months). This difference implied possible clinical benefit from ICIs beyond progression in selected populations after response to chemo‐immunotherapy for SCLC. Strikingly, median PFS, TTF, OS, and the median cycle of ICIs in 14 patients receiving ICIs beyond progression were 6.7 months, 21.4 months, 39.3 months, and 16 (range 5–42), respectively. These notable results suggested some cases could acquire an indolent tumor nature induced by continuous ICI exposure and obtain a favorable clinical course. One report indicated that OS in ED‐SCLC correlated more with post‐progression survival (PPS) than with PFS.^7^ Prolonged PPS associated with prolonged TTF could prolong OS in cases who benefited from continuous ICIs beyond progression.

Continuous ICIs beyond progression were administered to the following three relapse types: type 1, sensitive relapse as systemic rapid progression; type 2, isolated relapse; and type 3, sensitive relapse as systemic indolent progression. Type 1 treatment was being re‐treated with chemo‐immunotherapy; type 2, local ablative radiotherapy and continuous ICIs; and type 3, continuous ICIs alone. In SCLC, the efficacy of platinum rechallenge chemotherapy in sensitive relapse patients was reported.^8^ This study was conducted in the pre‐ICI era and it is unknown whether or not it can apply to the current ICI era. Our study included eight patients receiving platinum rechallenge with continuous ICIs and Figure 2 represents some clinical benefit from chemo‐immunotherapy rechallenge. The clinical courses of these patients suggest the possible effectiveness of platinum rechallenge with continuous ICIs in the current ICI era. Local ablative radiation therapy with continuous TKIs or ICIs beyond progression has shown their effectiveness in NSCLC.^6^, ^9^ A retrospective study reported that ED‐SCLC with oligometastases had a better prognosis than with polymetastases, and ED‐SCLC with oligometastases tended to recur locally.^10^ Nine patients in our study received local ablative radiation therapy with continuous ICIs and Figure 2 shows the long TTF in such cases. Since one report showed an abscopal effect with immunotherapy and radiotherapy in SCLC,^11^ we believe that this result was brought about by both the continuous administration of immunotherapy and local ablative radiation therapy. Thus, local ablative radiation therapy with continuous ICIs might also be useful, especially in oligoprogression of SCLC. Continuous ICIs with or without local ablative radiation therapy and/or platinum rechallenge could prolong TTF, resulting in PPS prolongation and thus OS.

In conclusion, our study suggests the effectiveness of continuous ICIs beyond progression in clinical practice for SCLC. The strategy could be suitably adopted and provide a favorable prognosis in selected cases of SCLC, which had been once regarded as an aggressive malignancy with generally pessimistic clinical courses. However, our study is retrospective with a small sample size, conducted at a single institution. To exclude various biases, further large prospective randomized studies are warranted to confirm the effectiveness of continuous ICIs beyond progression in SCLC.

## AUTHOR CONTRIBUTIONS

Ken Yamamoto: Project administration, conceptualization, methodology, formal analysis, investigation, data curation, writing – original draft/review and editing, visualization. Taira Ninomaru, Hideaki Okada, Katsuya Hirano, and Temiko Shimada: investigation, data curation, writing – original draft/review and editing. Akito Hata: Supervision, project administration, conceptualization, methodology, formal analysis, investigation, data curation, writing – original draft/review and editing, visualization.

## CONFLICT OF INTEREST STATEMENT

A.H.: honoraria, Eli Lilly, Chugai, Pfizer, Astrazeneca, Boehringer Ingelheim, Taiho; grants. M.S.D.: Eli Lilly, Boehringer Ingelheim, Astrazeneca. Other authors: no conflicts of interest.
